# The protein interaction map of bacteriophage lambda

**DOI:** 10.1186/1471-2180-11-213

**Published:** 2011-09-26

**Authors:** Seesandra V Rajagopala, Sherwood Casjens, Peter Uetz

**Affiliations:** 1 J Craig Venter Institute, Rockville, MD 20850, USA; 2 Division of Microbiology and Immunology, Pathology Department, University of Utah School of Medicine, Salt Lake City, UT 84112, USA; 3 Center for the Study of Biological Complexity, Virginia Commonwealth University, PO Box 842030, 1015 Floyd Ave., Richmond, VA 23284, USA; 4 Proteros Biostructures, Am Klopferspitz 19, D - 82152 Martinsried, Germany

## Abstract

**Background:**

Bacteriophage lambda is a model phage for most other dsDNA phages and has been studied for over 60 years. Although it is probably the best-characterized phage there are still about 20 poorly understood open reading frames in its 48-kb genome. For a complete understanding we need to know all interactions among its proteins. We have manually curated the lambda literature and compiled a total of 33 interactions that have been found among lambda proteins. We set out to find out how many protein-protein interactions remain to be found in this phage.

**Results:**

In order to map lambda's interactions, we have cloned 68 out of 73 lambda open reading frames (the "ORFeome") into Gateway vectors and systematically tested all proteins for interactions using exhaustive array-based yeast two-hybrid screens. These screens identified 97 interactions. We found 16 out of 30 previously published interactions (53%). We have also found at least 18 new plausible interactions among functionally related proteins. All previously found and new interactions are combined into structural and network models of phage lambda.

**Conclusions:**

Phage lambda serves as a benchmark for future studies of protein interactions among phage, viruses in general, or large protein assemblies. We conclude that we could not find all the known interactions because they require chaperones, post-translational modifications, or multiple proteins for their interactions. The lambda protein network connects 12 proteins of unknown function with well characterized proteins, which should shed light on the functional associations of these uncharacterized proteins.

## Background

**Figure 1:**
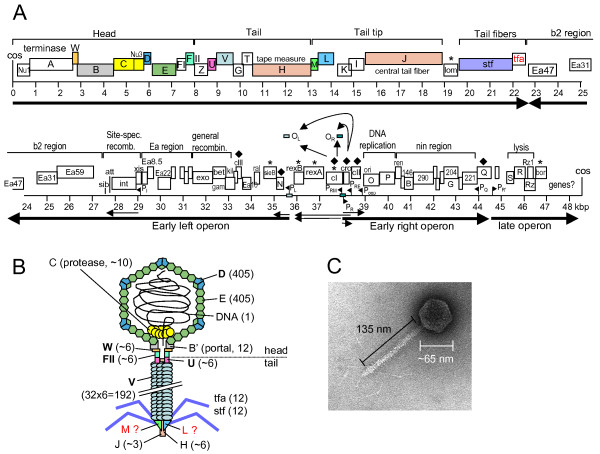
**The Lambda genome and virion**. **(A) **Genome of phage lambda. Colored ORFs correspond to colored proteins in (B). Main transcripts are shown as arrows. **(B) **A model of phage lambda, indicating protein-protein interactions. Proteins in bold font have known structures (Table 1). Numbers indicate the number of protein copies in the particle. It is unclear whether M and L proteins are in the final particle or only required for assembly. **(C) **Electron micrograph of phage lambda. (A) and (C) modified after [24].

Sixty years ago, in 1951, Esther Lederberg discovered phage lambda [1]. Since this seminal discovery lambda has become a model organism in which many foundational studies lead to our current understanding of how genes work and how they are regulated, as well as how proteins perform such functions as DNA replication, homologous and site-specific recombination, and virion assembly. In addition, tailed phages are the most abundant life form on earth [2], and so deserve to be studied in their own right and in the context of global ecology. Nevertheless, phage lambda is not completely understood. There are still a number of genes in its 48.5 kb genome whose function remains only vaguely defined, if at all. For instance, many of the genes in the b2 and *nin *regions have no known function (Figure 1). And 14 of the 73 predicted lambda proteins have unknown functions.

Two of the best-characterized aspects of lambda biology are the genetic switch that determines whether a phage reproduces and lyses the cell or whether it integrates into its host genomes to become a prophage [3,4] and the mechanisms through which transcription antitermination controls its gene expression cascade. Nevertheless, lambda continues to yield new insights into its gene regulatory circuits [4,5], and recent studies of its DNA packaging motor are in the vanguard of nanomotor research [6].

Surprisingly, even the structure of the lambda virion is incompletely known: the structures of only 5 of the ~14 proteins in the virus particle have been solved, and it is unknown whether several proteins that are required for tail assembly are in the completed virion, even though the overall structure is well known from electron microscopy [7].

Key to the understanding of lambda biology is a detailed understanding of protein function, including their interactions. We have curated more than 30 protein-protein interactions (PPIs) from the literature, identified over the past 60 years. Such interactions are reasonably well known within the virus particle and during the life cycle of lambda, i.e. during replication and recombination. However, the molecular details of virion assembly, obviously highly dependent on coordinated interactions of structural and accessory proteins, are still largely mysterious.

The structures of at least 17 lambda proteins have been solved (Table 1). In addition, the lambda head has been studied in some detail by cryo-electron microscopy, X-ray crystallography, and NMR (Figure 1). The tail is much less well known. While we do have structures of the head-tail junction proteins W, FII, and U individually, their connections to the head via the portal protein (B) and to each other are not very clear. Similarly, while we do have a structure of the major tail tube protein V, the remaining tail is structurally largely uncharacterized.

**Table 1:** Lambda proteins of known structure

Protein	PDB	reference
CI	3BDN	[77]
CII	1ZS4, 1XWR	[78,79]
Cro	2ECS, 2OVG, 2A63	[80,81]
D	1VD0, 1C5E, 1TCZ	[50,82,83]
Exo	1AVQ	[84]
FII	2KX4, 1K0H	[85,86]
Gam	2UUZ, 2UV1	[87]
Int	2WCC, 1P7D, 1Z19, 1Z1B, 1Z1G	[88-90]
N	1QFQ	[91]
NinB	1PC6	[26]
Nu1	1J9I	[33]
R	3D3D	[92]
NinI*	1G5B	[93]
U	3FZ2, 3FZB, 1Z1Z	[19,94]
V	2L04, 2K4Q	[94-96]
W	1HYW	[39]
Xis	2OG0, 2IEF, 1RH6, 1LX8	[69,97-99]

* Ser/Thr protein phosphatase

Our motivation for this study was three-fold: first, in our continuous attempts to improve the yeast two-hybrid system further, we thought that phage lambda would be an excellent "gold-standard" to benchmark our experimental system by demonstrating how many previously known interactions (Table 2) we are able to identify in such a well-studied system. Second, we believe that interaction data can help to solve the structures of protein complexes, since binary interactions as described here may facilitate the crystallization of co-complexes. Despite its well-understood biology, phage lambda is not well understood structurally; especially the assembly of its tail remains poorly understood. Third, and possibly most important, we wondered if we could contribute to the understanding of lambda biology, either by discovering new interactions or by verifying questionable or poorly supported interactions.

**Table 2:** Previously published interactions among lambda proteins

	interacting λ proteins		notes	ref#
**head**				

**1**	**A**	**Nu1**	A (N-term) - Nu1 (C-term)	[32-34]
2	A	B	A (C-term) - B (= portal)	[32,35]
**3**	**A**	**FI**	Genetic evidence	[21]
**4**	**FI**	**E**	Genetic evidence	[22]
5	Nu3	B	Nu3 required for B incorporation into procapsid	[36]
**6**	**W**	**B**		[37,38]
7	W	FII	W required for FII binding, FII connects head to tail	[37,39]
8	B	B	12-mer (22 aa removed from B N-term)	[40,41]
9	C	E	Covalent PPI (in virion?)	[42,43]
10	C	B		[44]
11	B	E	copurify in procapsid	[45]
**12**	**C**	**Nu3**	C may degrade Nu3 (before DNA packaging)	[45-47]
**13**	**D**	**D**	Capsid vertices, D forms trimers	[48-50]
**14**	**E**	**E**	Main capsid protein	[20,51,52]
**15**	**D**	**E**		[20,51,52]
	Nu3	Nu3	Nu3 multimer	unpublished *

**tail**				

**16**	**U**	**U**	"probably a hexamer", interact in crystal	[53]
17	V	V		[51,54-56]
**18**	**V**	**GT**	the T domain binds soluble V	[24]
**19**	**H**	**G/GT**	G/GT hold H in an extended fashion	[24]
**20**	**H**	**V**	V probably assembles around H, displacing G/GT	[57]

**replication**				

21	O	O	O-O interactions when bound to ori DNA	[58]
**22**	**O**	**P**		[59-62]

**transcription**				

**23**	**CI**	**CI**	Forms octamer that links O_R _to O_L_	[63,64]
**24**	**CII**	**CII**	homotetramers	[65]
25	CIII	CIII	dimer	[66]
26	Cro	Cro	dimer; x-ray structure	[67]

**Recombination**				

27	Exo	Bet		[68]
28	Xis	Int		[69] #
29	Xis	Xis	Xis-Xis binding mediates cooperative DNA-binding	[69] #
**30**	**Int**	**Int**	Dimer	[70]

**lysis**				

31	Rz	Rz1	heteromultimer that is supposed to span the periplasm	[71]
32	S	S	large ring in inner membrane	[72]
	S	S'	S' inhibits S ring formation (S: 105 aa, S': 107 aa)	[73]

**lysogenic conversion**				

33	SieB	Esc	Esc is encoded in frame in sieB + inhbits sieB	[74,75] #

**bold: **found in this study. * unpublished (C. Catalano, pers. comm., by permission), # interactions not tested in Y2H assays (one or both clones not available).

To achieve these goals, we cloned almost all lambda open reading frames (ORFs) and tested them for all pair-wise interactions, using a novel yeast two-hybrid strategy [8]. We identified a total of 97 unique interactions, most of which have not been previously described. About half of all published interactions were identified, and we will discuss why the other half has been missed and how these interactions might be detected by future two-hybrid studies.

## Results

### Approach

**Figure 2:**
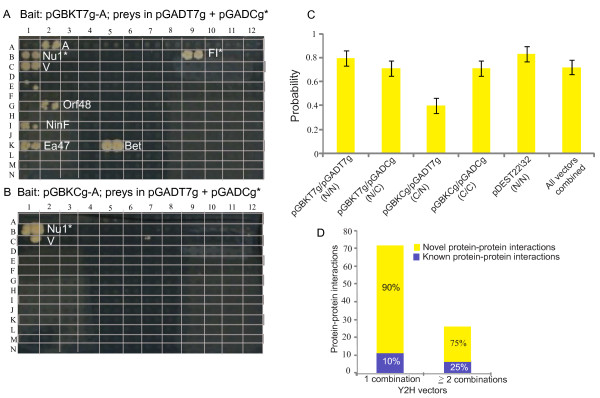
**Yeast two-hybrid array screens and vectors**. Shown are two Y2H screens with four different vector combinations. Each interaction is represented by two colonies to ensure reproducibility. **(A) **Lambda bait protein A (DNA packaging protein) was fused to an N-terminal DNA-binding domain ("DBD", in pGBKT7g) and was tested against prey constructs in both N- and C-terminal configurations (activation domains in pGADT7g, and pGADCg). **(B) **The C-terminal DBD fusion (in pGBKCg) as tested against prey constructs in both N- and C-terminal configurations (in pGADT7g, and pGADCg). The interactions of C-terminal preys are labeled with an asterisk (*), all remaining interactions use N-terminal fusions. All the interactions obtained from the array screening were subjected to Y2H retests: we were able to retest all the interactions shown in Figure 2 except A-Ea47, which has thus been removed from the final interaction list. Technical details of the screening procedure have been described in [8,10]. **(C) **Interaction quality assesment. Using the experimental derived false positive rate from [9] and Bayes theorem, we estimated the probability of an interaction to be true. This estimate depends on the vector system, being highest (83%) for pDEST22/32, and lowest (40%) for pGBKCg/pGADT7g. **(D) **Detection of known PPIs with different vector systems. Known PPIs are enriched in the subset of PPIs detected by > = 2 vector systems compared to PPIs detected by 1 vector combination.

In order to find as many interactions as possible, we cloned 68 lambda ORFs into six different Y2H vectors (see Table 3 and Methods). In fact, each vector pair results in very different subsets of interactions as we have shown previously [8-10]. For example, the pGADT7g/pGBKT7g vectors yielded 44 interactions while the pGBKCg/pGADCg vectors yielded only 18. The main difference between these two pairs is the way the fusion proteins are constructed: in the former two vectors the Gal4 DNA-binding (DBD) and activation domains (AD) are fused to the N-terminus of the lambda proteins (Figure 2). In the latter two the DBD and AD are fused to the C-terminus of the lambda proteins. It is thus reasonable to assume that structural constraints cause many of the observed differences.

**Table 3:** Vectors and interaction summary

Vector pair(s)	Fusions proteins	Interactions*
pDEST22/pDEST32	N/N (N-terminal fusions)	8
pGADT7g/pGBKT7g	N/N (N-terminal fusions)	44
pGBKT7g/pGADCg	N/C (N-terminal/C-terminal fusions)	39
pGBKCg/pGADCg	C/C (C-terminal/C-terminal fusions)	18
pGBKCg/pGADT7g	C/N (C-terminal/N-terminal fusions)	26

* Redundant, i.e. some interactions are found with multiple vectors.

Fusion proteins indicate the location of the DNA-binding (DBD) and activation domains (AD), respectively, of each vector pair. For instance, the pDEST vectors both have the DBD and AD fused at the N-terminus of the bait and prey protein. Vectors are listed as bait/prey pairs.

### Assay sensitivity and false positives

As we have observed before in other contexts [10], the pGADT7g/pGBKT7g vectors yielded almost half of all interactions discovered in this study and almost three times as many as the pDEST series of vectors (which uses similar N-terminal fusions). The pDEST system may detect fewer interactions but they probably also detect fewer false positives (see discussion).

In a previous study we benchmarked the false positive rate for each Y2H vector systems under different screening (stringency) conditions [9]. To evaluate the accuracy of the vector system we used specificity estimates from this study [9] (i.e., the experimental proportions of negative interactions among negative reference interactions). The sensitivity was estimated using known lambda interactions (i.e., the experimental proportion of positive interactions among positive reference interactions). Specificity ranged from most specific, namely 98.9% for GADT7g/pGBKT7g and pGBKT7g/pGADCg to 95.7% for pGBKCg/pGADT7g (least specific). Sensitivity ranged from 33.3% for pGBKT7g/pGADCg to 17% for pGBKCg/pGADCg and pDEST22/32. For each method, we estimated the probability of being a true interaction using Bayes theorem: pDEST22/32 (83.3%), pGADT7g/pGBKT7g (80.0%), pGBKT7g/pGADCg and pGBKCg/pGADCg (71.4%), and pGBKCg/pGADT7g (40.0%) (Figure 2C).

### Verification and quality scores

If an interaction is found in more than one vector combination, the reliability is higher than when it is found in only one. Twenty-four interactions (out of 97) were found in 2 or more vector combinations (Table 4). This number of combinations can be used as a score, and the 3 interactions with the highest score have all been described in the literature before. Of the 24 high-scoring interactions, six (25%) have been described before (Figure 2D). To test if the difference of the proportions of detected literature interactions is greater for the more than one vector combination group, we carried out a one-sided test for difference of proportions. The null hypothesis can be rejected for alpha = 0.1 indicating a moderately significant difference (P-Value = 0.098) (Additional file 1: **Table S6**). We conclude that the number of supporting vector combinations can be used as a confidence score. This suggests that the 18 novel high-scoring interactions are possibly physiologically relevant interactions and thus good candidates for further studies (see discussion).

**Table 4:** All PPIs discovered in this study

	Bait	Prey	Bfun	Pfun	NN	NC	CC	CN	Vectors	Notes
1.	A	A	head	head		NC	CC	CN	3	Possible
2.	A	Bet	head	rec	G				1	
3.	A	FI	head	head		NC	CC'	CN'	3	Known
4.	A	NinF	head	ukn	G				1	
5.	A	Nu1	head	head	G'	**NC'**	CC		3	Known
6.	A	Orf79	head	unk	G				1	
7.	A	V	head	tail	G				1	
8.	Cl	Cl	trx	trx			CC		1	Known
9.	Cl	Kil	trx	other			CC		1	
10.	Cll	Cll	trx	trx		NC			1	known
11.	C	C	head	head		NC			1	
12.	C	Nu3	head	head	G'	**NC'**			2	Known
13.	C	Orf79	head	unk	G				1	
14.	D	D	head	head		NC			1	Known
15.	D	E	head	head	D				1	Known
16.	E	E	head	head	D				1	Known
17.	E	Fi	head	head	G	NC	CC'	CN'	4	Known
18.	E	Nu3	head	head	DG'				2	2v
19.	Ea8.5	Ea8.5	ihr	unk		NC			1	Possible
20.	Ea8.5	Int	ihr	rec	G	NC			2	2v
21.	Ea8.5	Tfa	ihr	tail	G				1	
22.	Ea8.5	Stf	ihr	tail	G			CN	2	
23.	Ea8.5	Q	ihr	trx	G				1	
24.	Ea8.5	Ren	ihr	unk		NC			1	
25.	FI	NinB	head	rec				CN	1	
26.	G	G	tail	tail	G		CC	CN	3	Possible
27.	G	H	tail	tail	**D'**				1	Known
28.	G	S'	tail	lysis	G			CN	2	2v
29.	G	T	tail	tail			CC		1	Likely
30.	H	Cll	tail	trx		NC			1	
31.	H	Ren	tail	unk		NC			1	
32.	H	V	tail	tail		NC			1	Known
33.	Int	A	rec	head	G				1	
34.	Int	Bet	rec	rec	G				1	Possible
35.	Int	Int	rec	rec	G	NC			2	known
36.	Int	Orf48	rec	unk	G				1	
37.	Int	Tfa	rec	tail				CN	1	
38.	Int	V	rec	tail	G				1	
39.	M	Fi	tail	head			CC'	**CN'**	2	2v
40.	M	G	tail	tail	G		CC	CN	3	Possible
41.	M	NinF	tail	unk	G			CN	2	2v
42.	M	Nu3	tail	head				CN	1	
43.	M	Orf35	tail	unk		NC	CC		2	2v
44.	N	Bet	trx	rec	G				1	
45.	N	Ea47	trx	unk	G				1	
46.	N	L	trx	tail	G				1	
47.	N	Nu1	trx	head		NC			1	
48.	N	V	trx	tail	G				1	
49.	NinD	Cro	unk	trx	G				1	
50.	NinD	K	unk	tail	G	NC			2	2v
51.	NinD	Q	unk	trx	G				1	
52.	NinI	N	unk	trx	G				1	
53.	NinI	Q	unk	trx	G				1	
54.	Nu1	Nu1	head	head		NC	CC		2	2v
55.	Nu1	Tfa	head	tail	G				1	
56.	Nu1	Orf64	head	unk			CC		1	
57.	Nu1	R	head	lysis	D				1	
58.	Nu1	V	head	tail	G				1	
59.	Nu3	Nu3	head	head	G				1	
60.	Nu3	Z	head	tail	G				1	
61.	O	P	repl	repl	D				1	Known
62.	Orf35	Cll	unk	trx		NC			1	
63.	Orf35	Int	unk	rec	G	NC			2	2v
64.	Orf35	K	unk	tail	G	NC			2	2v
65.	Orf35	Orf78	unk	unk		NC			1	
66.	Orf35	Ren	unk	unk		NC			1	
67.	Orf48	Orf48	unk	unk		NC			1	Possible
68.	Orf79	Orf79	unk	unk			CC	CN	2	Possible
69.	Orf63	N	rec	trx	G				1	
70.	Orf63	Orf78	rec	unk		NC			1	
71.	Orf63	P	rec	repl		NC			1	
72.	Orf63	Q	rec	trx	G				1	
73.	Orf63	Ren	rec	unk		NC			1	
74.	Orf63	Rz1	rec	lysis	G				1	
75.	P	Bet	repl	rec	G				1	
76.	P	Q	repl	trx	G				1	
77.	RexB	A	conv	head		NC			1	
78.	RexB	Orf48	conv	unk		NC			1	
79.	RexB	Orf78	conv	unk		NC			1	
80.	RexB	Ren	conv	unk		NC			1	
81.	S'	S'	lysis	lysis	G				1	
82.	U	Ea47	tail	unk			CC	CN	2	2v
83.	U	NinB	tail	rec				CN	1	
84.	U	NinE	tail	unk				CN	1	
85.	U	NinF	tail	unk				CN	1	
86.	U	Orf78	tail	unk		NC			1	
87.	U	U	tail	tail			CC		1	known
88.	U	Xis	tail	rec		NC			1	
89.	V	G	tail	tail	D	NC			2	Known
90.	W	B	head	head		NC			1	Known
91.	U	Cl	tail	trx				CN	1	
92.	M	Rz1	tail	lysis			CC	CN	2	2v
93.	Orf79	NinB	unk	rec				CN	1	
94.	Int	G	rec	tail	G			CN	2	2v
95.	Ea.85	NinB	unk	rec				CN	1	
96.	S'	NinB	lysis	rec				CN	1	
97.	S'	Rz1	lysis	lysis				CN	1	

**Bfun **= bait protein function, **Pfun **= prey protein function group (**rec **= recombination, **repl **= replication, **trx **= transcription, **conv **= lysogenic conversion, **ihr **- inhibition of host replication [76]). NN, CN, NC, CC indicated the fusion type of the bait and prey proteins (see text). The two NN vectors are indicated by G (pGBK/pGAD) and D (pDEST22/32). Interaction that have been found in inverted prey-bait combinations are indicated by a prime sign ('). Interactions that have been found in both bait-prey and prey-bait orientations are indicated by bold and primes (e.g. **NC'**), respectively. Interactions without any note are unexpected and may not be physiologically relevant. 2v = interactions found with 2 vector pairs. Stf = Orf314.

Of the 73 interactions that were found in only one combination, 10 have been published previously, demonstrating that they are useful too. In fact, 16 out of 30 previously found interactions were also found in our screen, i.e. 53%. Note that three previously found interactions (Xis-Xis, Xis-Int, and SieB-Esc) could not be tested since we were unable to obtain ORF clones of J, Xis, NinH, and Esc (which is encoded within SieB).

### Prey counts

There are other criteria that can be used to score interactions. One of them is the number of times a prey protein is found. This "prey count" indicates whether a protein interacts very specifically (low prey count) or more unspecifically and thus promiscously. Proteins with high prey counts are more likely false positives, and hence we removed these interactions with prey count > 5 from further analysis (see Additional file 1: **Tables S2 **and **S3**). However, this was not generally true in our study: of the preys that were found 1 to 3 times, 12 were found among the "gold-standard" literature interactions. Of the preys that were found 4 to 5 times, 9 were involved in such gold-standard interactions (5 interactions were shared in both groups).

### Protein coverage

**Figure 3:**
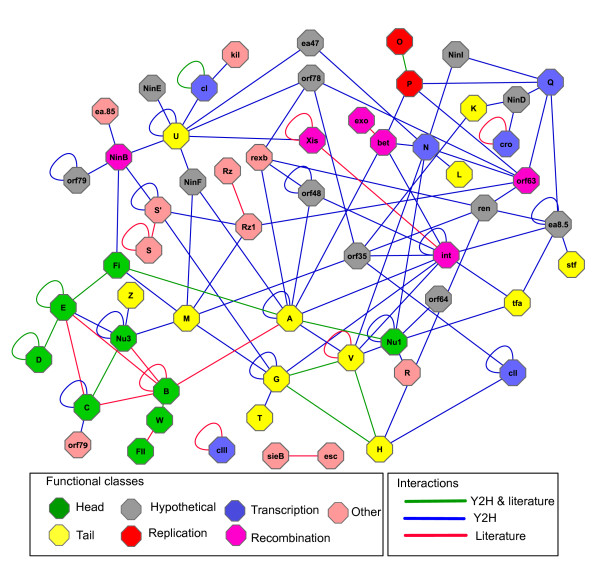
**The protein interaction network of phage lambda**. Interactions from this study have been integrated with previously published interactions ("literature"). Nodes in the network represent proteins and are colored according to their functional class (see color key). The protein-protein interactions are indicated by lines ("edges"). The edge color represents the source of the interactions, e.g., all red edges are previously reported interactions, all blue interactions were identified in our two-hybrid study, and all green interactions are previously known and are reproduced in our study.

Among the 73 lambda proteins listed in the Uniprot database (J02459), 51 were found to be involved in interactions (Figure 3), which represents 70% of the proteome. 15 proteins were found only in one interaction (CIII, Ea10, Ea59, Exo, FII, Kil, L, Nu3, Orf64, Orf60a, R, Rz, T, W, and Xis) but 7 proteins were found to be involved in 10 or more interactions (namely U, Bet, Ea8.5, Nu1, A, Int, and G). Hence the former are more specific and latter more promiscous and thus less reliable. Interestingly, several proteins were conspicuously absent from our list of interactions, primarily proteins of head and tail assembly (B, C, I, J, Stf, and Tfa) as well as the poorly understood proteins NinG, NinH, Orf221 (NinI), Orf290 (NinC), and SieB (see discussion).

### Functional specificity

**Figure 4:**
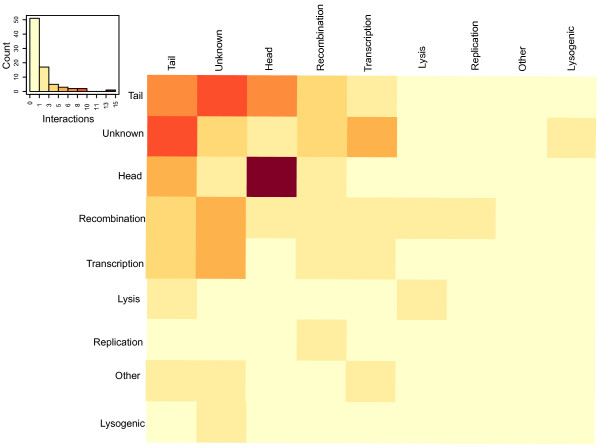
**Interactions among functional groups of proteins**. Each row and column of the shown profile corresponds to a protein-protein interaction (two-hybrid) count with different functional classes (see matrix). The interactions within certain functional classes are enriched compared to other functions groups, e.g. head assembly proteins show 15 interactions among each other, 8 interactions are detected between tail assembly proteins and 3 interactions among proteins of unknown function (see Additional file 1: **Tables S4 **and **S5 **for details).

We grouped all lambda proteins in 9 groups, namely virion head, virion tail, transcription, replication, recombination, lysis, lysogenic conversion, others with known function, and unknown (Table 4). A statistical analysis of interactions shows that proteins involved in head assembly have the highest specificity (Figure 4): when interactions among different functional classes are considered, the proteins involved in capsid assembly tend to interact with themselves more frequently compared to other functional classes. Interestingly, the proteins of unknown function show interactions with proteins involved in several functional classes, including tail assembly, transcription and recombination (Figure 4).

Overall, the 97 protein-protein interactions (PPIs) of our screens correspond to ~4.2% of the lambda search space (= 97/68*68*0.5), i.e. all possible protein pairs of the lambda proteome (here: 68*68). This is significantly less than we found in *Streptococcus *phage Dp1, namely 156 interactions among 72 ORFs [11] even though in the latter case only 2 vector pairs were used. A possible explanation is that we used a more rigorous retesting scheme here in which only interactions were counted that were found in multiple rounds of retesting.

## Discussion

### Lambda protein interaction network

This is only the second study that has applied multiple two-hybrid vector systems to characterize the protein-protein interactions at a genome scale, the first being our analysis of the Varicella Zoster Virus [8]. The lambda protein network connects 12 proteins of unknown function with well characterized proteins, which should shed light on the functional associations of these uncharacterized proteins (Figure 3). For example, NinI interacts with two proteins N and Q which are involved in transcription antitermination. The scaffolding protein Nu3 forms dimers, and interacts with the tail proteins Z and M as well as the capsid protein E. Thus, Nu3 may play an accessory role in the assembly of both head and tail, even though Nu3 is not absolutely required for tail assembly.

### False negatives

This study discovered more than 53% of all published interactions among lambda proteins. However, it failed to discover the remaining 47%. We can only speculate why this is the case. Some of the early steps in virion assembly depend on chaperones [12]. For instance, the portal protein B requires GroES/EL, most likely for folding [13]. These chaperones are not present in the yeast cells which we used for our interaction screens. We found only one of five known interactions of B (namely W-B) and aberrant folding in yeast may be the reason for not detecting the other four known interactions. In addition, several lambda proteins are processed during assembly. For instance, the C protease is processed and covalently linked to the capsid protein E. This fusion protein is then further processed to yield products named "X1" and "X2" even though recent attempts to identify X1 and X2 were unsuccessful and thus X1 and X2 may be artifacts [14]. A 21 amino acid peptide is also proteolytically removed from the portal protein B but it is not known how this affects its interaction properties. Finally, protein S, which forms a membrane protein involved in lysis, is made in two variants that use different start codons. In fact, we do find that the shorter variant, S' (105 amino acids) has a slightly different interaction pattern compared to the full-length variant, S (107 amino acids) (Figure 3). We have not investigated the detailed mechanism of these differences but it has been shown in several studies that fragments of proteins show different interaction patterns than their full-length proteins [15,16] even though this is an extreme case given the small difference between S and S'. While sterical hindrance may be an obvious reason for this behavior, little is known about the mechanistic details in most other published cases.

False negatives may also be a result of the obligate stepwise assembly of large protein structures in lambda and other phage, e.g. when a conformational change due to interaction between two proteins creates a new binding site for a third protein. For instance, in phage T7 only the heterodimer of gp5 and the host thioredoxin provides a binding site for the single-stranded-binding protein (SSB = gp2.5) and the primase-helicase gp4 [17]. Such cases can only be detected if all three proteins were expressed simultaneously and the constructs involved allowed the formation of complex oligomers.

### False positives

While we found only 53% of all previously known interactions of lambda, we also found many new ones (Table 4). However, many of the new interactions have only been found once and hence are lower confidence interactions. On the other hand, nine of the previously published interactions were found only once in our screen but are nevertheless well-known interactions. In order to verify the biological significance of new interactions further criteria or experiments are required. One criterion often used is the plausibility of an interaction: if two interacting proteins belong to the same functional group, they are likely physiological. 34 of the 97 interactions (34%) take place within their functional group, including the 16 known ones. Some of the remaining interactions are discussed below in the context of their functional group.

Some proteins appear to be particularly "sticky". For example, G, a tail protein, is involved in 8 different two-hybrid interactions. The specificity of such interactions is inversely proportional to the number of such interactions; thus, G likely interacts rather unspecifically, and its interactions have to be interpreted cautiously. Similarly, Int, A, Nu1, and U are involved in 8 or more two-hybrid interactions each, and thus each interaction has to be evaluated individually keeping in mind its promiscuity. We have attempted a careful manual evaluation in Table 4.

The reason for interaction promiscuity and thus false positives remains unclear. Several hypotheses have been proposed to explain such cases. For example, a protein may have hydrophobic patches that interact unspecifically. Some authors have suggested that simply an increase in abundance might cause a promiscuous gain of interactions [18] but such theories remain to be tested rigorously.

The Y2H assay appears to be sensitive enough to detect weak interactions that are not detectable in NMR experiments, e.g. the interaction between U monomers [19]. The high sensitivity may also explain a significant number of false positives which may have been detected in our screen but which do not have any physiological significance. Future quantitative measurements are thus required to clarify the relationship between affinity and physiological relevance.

### Head assembly and structure

**Figure 5:**
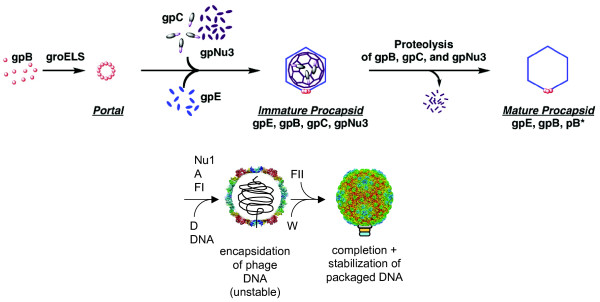
**Head assembly**. Head assembly has been subdivided in five steps although most steps are not very well understood in mechanistic terms. The tail is assembled independently. The C protease, the scaffolding protein Nu3, and the portal protein (B) form an ill-defined initiator structure. Protein E joins this complex but the chaperonins GroES and GroEL are required for that step. Within the prohead C and E are processed to form covalently joined X1 and X2 proteins although this is controversial (see text). Proteins Nu1, A, and FI are required for DNA packaging. Protein D joins and stabilizes the capsid as a structural protein. FII and W are connecting the head to the tail that joins once the head is completed. Modified after [12] and [20].

The structure of the lambda protein shell is known in great detail [20]. However, its assembly is much less well understood as are the locations and functions of the "minor" proteins that are present in only a few molecules/virion (Figure 5). The portal protein B is believed to be the nucleator or initiator of head assembly, which first assembles with the C protease and with the scaffolding protein Nu3 into an ill-defined initiator structure. B, C, and Nu3 are known to form a complex in which several interactions have been previously reported (C'-B, C-Nu3, Nu3-Nu3, and Nu3-B, Table 2). We could not detect B in any interaction although we did find Nu3-C, Nu3-Nu3 and Nu3 interactions with E and Z. This is noteworthy because Nu3-E and Nu3-Z are new interactions. It is known that E (the major capsid protein) assembles onto or around the initiator structure to form the procapsid [12], and it is conveivable that B joins such an assembly. If Nu3 and C proteins are both required for B to join, we would have missed this interaction, given that we tested only pairs of proteins. Nu3 also appears to form dimers by the Y2H analysis, and this has been confirmed independently (C. Catalano, pers. comm.).

The head shell is bound by the D protein which stabilizes the coat protein shell. However, if Nu1, A, or FI are missing, DNA is not packaged and as a consequence, the coat shell does not expand, and D can only add after expansion. We could confirm the A-Nu1 interaction as well as the interactions between FI and A and FI and E which were previously known only from genetic experiments [21,22]. We also confirmed the D-E and E-E interactions.

The terminase and the portal proteins are the largest proteins of the lambda head. Using fragments of these proteins as baits - as opposed to full-length proteins - may result in additional interactions, especially since we were not able to detect most of the B interactions reported in the literature (Tables 2 and 4).

### Tail assembly and structure

**Figure 6:**
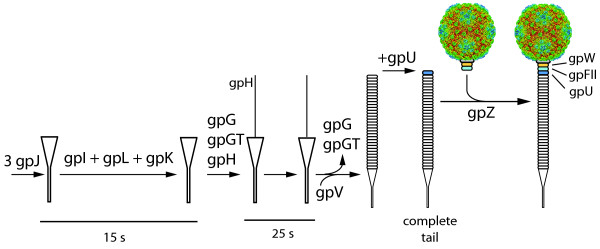
**Tail assembly**. The lambda tail is made of at least 6 proteins (U, V, J, H, Tfa, Stf) with another 7 required for assembly (I, M, L, K, G/T, Z). Assembly starts with protein J, which then, in a poorly characterized fashion, recruits proteins I, L, K, and G/T to add the tape measure protein H. G and G/T then leave the complex so that the main tail protein (V) can assemble on the J/H scaffold. Finally, U is added to the head-proximal end of the tail. Protein Z is required to connect the tail to the pre-assembled head. Protein H is cleaved between the action of U and Z [31]. It remains unclear if proteins M and L are part of the final particle [24]. Modified after [23].

Tail assembly is even less well understood than head assembly (Figure 6). From genetic analyses it is known that the host receptor protein J initiates the process with I, L, K, and G (including its fusion protein G-T) successively joining the process [23]. Older studies suggest a slightly different order of action, namely J > I > K > L [24]. In fact, it is not known if I, L and M are components of the finished virion or are assembly factors that are not present in virions. It is thus difficult to reconstruct the detailed molecular events during tail assembly. In any case, J eventually associates with the tape measure protein H, and the major tail protein V forms a tube around this central rod. U finally joins the head-proximal part of the tail. Similarly, W and FII join to the portal protein in the head to form the binding site for the tail. The main tail proteins are connected by known direct protein-protein interactions (Table 2) but the interactions during the initiation of tail assembly have eluded previous studies. In fact, we failed to detect any interaction involving J and I, and the only interactions of L and K did not involve other tail proteins (Table 4). However, we did find several new interactions that are potentially relevant for tail assembly. For instance, G, a fairly promiscous protein with a total of 8 interactions, was found to bind to V, G, T, H, and M. It is thus possible that it acts as a scaffold organizing the assembly of the tail. By contrast, the interactions of H and V with G were their sole tail-related interactions. We did not find the tail fiber proteins Stf and Tfa to interact with other tail proteins in our screens. Stf has been speculated to assume a trimeric structure, similar to the tail fiber protein of phage T4 [25] although there is no specific evidence for oligomerization in lambda.

In summary, it is surprising that we found so many virion protein interactions, given that virion assembly is an obligately ordered pathway and most binding sites may be only present in the growing virion and not on individual unassembled proteins.

### Transcription

The genetic switch leading to a decision between lysogeny and lysis has made lambda a prime model system for transcriptional regulation. A significant fraction of lambda literature has been devoted to this question [3]. Here, we ignore the interactions of transcription factors with DNA and concentrate on their interactions among each other and the transcriptional machinery. Several factors form dimers (Cro, CI, CII, CIII). Of these, we could only confirm the CII self-interaction. CI, CII, and CIII all interact with various components of the virion in our two-hybrid studies, especially of the tail. However, whether these interactions are physiologically relevant is questionable. Notably, the antiterminators N and Q also show a number of interactions in our tests although none of these involve any other transcriptional regulators. Also, all of these interactions were found in a single vector combination, so they are not as well supported as other interactions.

### Recombination, integration, and excision

Integration of the lambda genome into the host chromosome is part of the establishment of the lysogenic state. Integrase (Int), assisted by the integration host factor (IHF) catalyzes this reaction. Similarly, integrase (Int), this time assisted by excisionase (Xis) and the host Fis protein, catalyzes the excision of the lambda prophage. Three other lambda proteins are known to be involved in homologous recombination: Exo (exonuclease), Bet (= β, strand annealing protein), Gam (an anti-recBCD protein), and NinB (which can replace the recFOR complex which can load RecA onto ssDNA covered with single-stranded DNA-binding (SSB) protein [26]). We did not find the known interaction between Bet and Exo. In fact, we found Int and Bet to both homodimerize, and Bet and Int to interact. This indicates that these proteins may assist Int. A number of other interactions involving these recombination proteins and unrelated gene products are difficult to explain and require further analysis. However, they may implicate several uncharacterized small ORFs in the process of recombination (Table 4).

### Host interactions

At least 15 lambda proteins interact with host proteins (S. Blasche, S.V. Rajagopala & P. Uetz, unpublished data). Lambda critically depends on host factors for integration, transcription, excision and virion assembly. Hence, a detailed understanding of lambda biology depends on information about such host-phage interactions. These interactions are beyond the scope of this study. We will address this issue in a forthcoming paper.

### Protein networks and functional genomics of phage lambda

Phage lambda has been studied almost exclusively by detailed and directed functional studies for the past 60 years. Systematic or large-scale studies have been initiated only recently. For instance, Maynard et al. [27] have screened the KEIO collection of *E. coli *deletion mutants for genes that affect lambda reproduction. This study found 57 *E. coli *genes of which more than half had not been associated with lambda biology before. Similarly, Osterhout et al. [28] investigated *E. coli *gene expression as a result of prophage induction and found 728 genes to change their expression patterns when lambda lysogens are induced. We expect to finish our own screens of lambda-host interactions soon and integrate the resulting protein-protein interactions into a systems biology model of lambda biology.

## Conclusions

Using phage lambda as a benchmark we showed that we can find about 50% of the interactions among its proteins using Y2H screens. No other technology has been able to detect such a large fraction of interactions in a single macromolecular assembly (except crystallization of whole complexes, which is not possible with phage particles). We thus predict that our strategy can find roughly half of all interactions in other phage and protein complexes. However, other methods will be required to find interactions that require chaperones, post-translational modifications, or other additional factors that could not be provided in our assay.

## Methods

### Cloning the phage lambda ORFs into Gateway entry vector

The DNA sequence of phage lambda was obtained from the NCBI genomes database (NC_001416) and primers were designed, using the Primer Design Tool [29]. The primers were designed without endogenous stop codons. In addition to the 20- to 30-nucleotide-long ORF-specific sequence the *attB1 *segment (5'-aaaaagcaggctta-3') was added to each forward primer, followed by ORF-specific bases. The *attB2 *segment (5'-agaaagctgggtg-3') was added at the 5' end of each reverse primer, which was complementary to the end of the ORF, without the last nucleotides of the stop codon.

### PCR amplification and cloning of lambda ORFs into gateway entry vector

All the ORFs of phage lambda were PCR amplified using KOD DNA polymerase (Novagen), and phage lambda genomic DNA (NEB:N3011L). The complete sequences of attB1 (5'-GGGGACAAGTTTGTACAAAAAAGCAGGCT-3') and attB2 (5'-GGGGACCACTTTGTACAAGAAAGCTGGGT-3') were added in the secondary round PCR, where the first round PCR product was used as a template, to generate the full-length attB1 and attB2 sites flanking the ORFs. The PCR cycles were used as recommended by the KOD DNA polymerase manufacturer (Novagen, Cat. No.710853).

The PCR-amplified ORFs with attB1 and attB2 sites were recombined into the entry vector pDONR™/Zeo (Invitrogen) by using the BP Clonase™ II Enzyme Mix (Invitrogen). The products resulting from site-specific recombination were transformed into chemically competent *E. coli *(DH5-α) and plated onto solid LB medium containing Zeocin. Two isolated colonies were selected for each reaction and the clones were verified by colony-PCR with pDONR™/Zeo-specific primers. The clones that had an insert of the expected size were picked for plasmid isolation and the plasmid preparations were sequenced with a pDONR™/Zeo-specific forward and reverse primers to verify the insert from both N-terminal and C-terminal ends of the ORFs. All the sequencing reads were analyzed using NCBI standalone BLAST against the phage lambda genome to confirm the identity of each ORF. We obtained 68 entry clones out of 73 targeted lambda ORFs (see Additional file 1: **Table S1**).

### Yeast two-hybrid clones

All the lambda phage ORFs in the entry vectors are sub-cloned into yeast two-hybrid expression vectors (Table 3), by using the LR Clonase™ II Enzyme Mix (Invitrogen). The destination vectors used were pDEST22, pDEST32 (Invitrogen), pGADT7g, pGBKT7g and pGADCg, pGBKCg vectors [8].

### Yeast two-hybrid screening

We carried out comprehensive Y2H interaction screening with the following Y2H vector pairs: pDEST32-pDEST22, pGBKT7g-pGADT7g, pGBKT7g-pGADCg, pGBKCg-pGADCg and pGBKCg-pGADT7g (listed as bait-prey vector pair). In the array screening we tested each protein both as activation (prey) and DNA-binding domain fusion (bait), including C-terminal fusions in pGBKCg and pGADCg. This way, we tested each protein pair in ten different configurations (Figure 2). The yeast two-hybrid assays were conducted as described in detail by Rajagopala et al. [10,30].

#### Data availability

The protein interactions from this publication have been submitted to the IMEx http://www.imexconsortium.org consortium through IntAct http://www.ebi.ac.uk/intact/ and assigned the identifier IM-15871.

## Authors' contributions

SVR conducted all experiments and analyzed data. SRC analyzed data and wrote part of the paper. PU conceived this study, analyzed data, and wrote part of the paper. All authors contributed in writing the manuscript and approved its final content.

## Supplementary Material

Additional file 1 **Tables S1-S7(Excel spreadsheet with tables in individual sheets)**. S1. Lambda pDONR clones. S2. Lambda protein-protein interactions from Y2H screening. S3. Lambda protein-protein interactions with high prey count (unspecific interactions). S4. Phage Lambda Genome Anotation (Uniprot). S5. Protein interaction with different functional groups. S6. Protein interaction confidence assessment. S7. Layout of Y2H preys pGADT7g and pGADC on screening plates. Click here for file
